# Regulatory role of NKG2D+ NK cells in intestinal lamina propria by secreting double-edged Th1 cytokines in ulcerative colitis

**DOI:** 10.18632/oncotarget.22132

**Published:** 2017-10-30

**Authors:** Fan Wang, Pai-Lan Peng, Xue Lin, Ying Chang, Jing Liu, Rui Zhou, Jia-Yan Nie, Wei-Guo Dong, Qiu Zhao, Jin Li

**Affiliations:** ^1^ Department of Gastroenterology, Zhongnan Hospital of Wuhan University, Wuhan 430071, China; ^2^ Department of Gastroenterology, Renmin Hospital of Wuhan University, Wuhan 430060, China; ^3^ Hubei Clinical Center & Key Laboratory of Intestinal & Colorectal Diseases, Wuhan 430071, China; ^4^ Department of Gastroenterology, The Central Hospital of Enshi Autonomous Prefecture, Enshi 445000 China; ^5^ Department of Critical Care Medicine, The People's Hospital of Huangshan, Huangshan 245000 China

**Keywords:** intestinal lamina propria, NKG2D, natural killer cells, ulcerative colitis

## Abstract

The role of intestinal lamina propria (LP) NKG2D+ NK cells is unclear in regulating Th1/Th2 balance in ulcerative colitis (UC). In this study, we investigated the frequency of LP NKG2D+ NK cells in DSS-induced colitis model and intestinal mucosal samples of UC patients, as well as the secretion of Th1/Th2/Th17 cytokines in NK cell lines after MICA stimulation. The role of Th1 cytokines in UC was validated by bioinformatics analysis. We found that DSS-induced colitis in mice was characterized by a Th2-mediated process. In acute phrase, the frequency of LP NKG2D+ lymphocytes increased significantly and decreased in remission, while the frequency of LP NKG2D+ NK cells decreased significantly in acute phase and increased in remission. No obvious change was found in the frequency of total LP NK cells. Similarly, severe UC patients had a higher expression of mucosal NKG2D and a lower number of NKG2D+ NK cells than mild to moderate UC. In NK cell lines, the MICA stimulation could induce a predominant secretion of Th1 cytokines (TNF, IFN-γ). Furthermore, in bioinformatics analysis, mucosal Th1 cytokine of TNF, showed a double-edged role in UC when compared to the Th1-mediated disease of Crohn's colitis. In conclusion, LP NKG2D+ NK cells partially played a regulatory role in UC through secreting Th1 cytokines to regulate the Th2-predominant Th1/Th2 imbalance, despite of the concomitant pro-inflammatory effects of Th1 cytokines.

## INTRODUCTION

Inflammatory bowel diseases (IBD) are chronic inflammatory disorders of the gastrointestinal tract, including ulcerative colitis (UC) and Crohn's disease (CD) [1]. The incidence was steadily on the rise during the past decades [2]. Mucosal immune dysfunction was thought to induce the inflammation by activating both adaptive and innate immunity [3]. In adaptive immunity, CD was predominated by T helper (Th) 1 and Th17 cells, while UC was characterized by a Th2 disorder [4]. In innate immunity, several subtypes of mucosal natural killer (NK) cells and their secreted cytokines were found to be involved in the pathogenesis of IBD [5–7]. As one of the main activated receptors in NK cells, NKG2D (natural killer group 2 member D) and its ligand MICA (MHC class I-related chain molecules A) were up-regulated in UC mucosa, and associated with the disease activity [8, 9]. However, the role of mucosal NKG2D+ NK cell in UC was unknown. Thus, we focused on lamina propria NKG2D+ NK cells, and attempted to clarify its role in the pathogenesis of UC.

## RESULTS

### DSS-induced colitis in mice and disease severity

**Figure 1:**
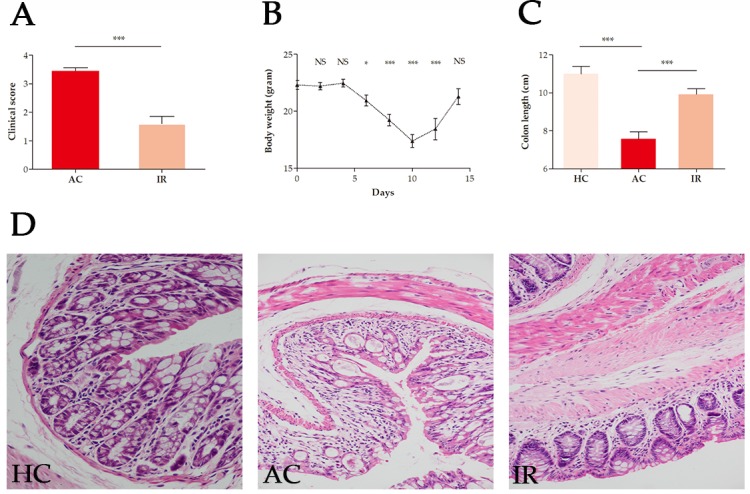
Evaluation of disease severity during DSS-induced colitis in mice **(A)** Clinical scores. **(B)** Body weight. **(C)** Colon length. **(D)** Histologic characteristics (200-fold image magnification). AC: acute colitis, IR: in remission, HC: healthy controls, ^*^: *P* < 0.05, ^**^: *P* < 0.01, ^***^: *P* < 0.001, NS: not significant.

Mice were given 4.5% DSS for 7 days to induce acute colitis (AC). On day 3, they started to develop clinical symptoms, such as diarrhea, hematochezia and body weight loss (Figure 1). After discontinuation of DSS administration for another 7 days, the mice were in remission (IR).

### Production of pro-inflammatory cytokines during DSS-induced colitis in mice

**Figure 2:**
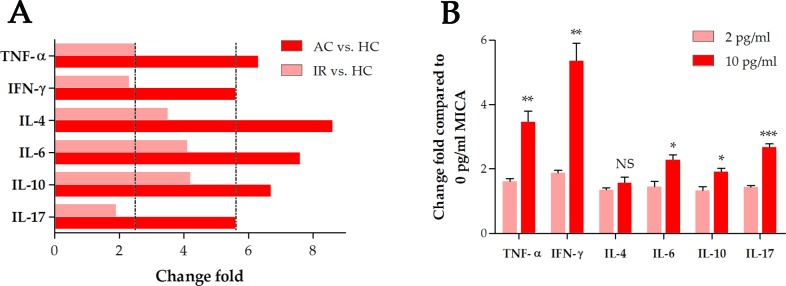
Change fold of cytokines **(A)** Change fold of intestinal cytokines in DSS-induced colitis. **(B)** Change fold of mRNA levels of Th1/Th2/Th17 cytokines in NK-92 cell lines after MICA stimulation at different concentrations. AC: acute colitis, IR: in remission, HC: healthy controls, TNF: tumor necrosis factor, IFN: interferon, IL: interleukin, ^*^: *P* < 0.05, ^**^: *P* < 0.01, ^***^: *P* < 0.001, NS: not significant.

To assess the degree of inflammation, we tested the Th1 (TNF-α, IFN-γ), Th2 (IL-4, IL-6, IL-10) and Th17 (IL-17) cytokines in intestinal tissues. The production of cytokines was increased significantly in acute colitis (*P* < 0.05), and decreased significantly in remission (Figure 2A). Th2 cytokines had a remarkably higher change fold than Th1/Th17 cytokines in both AC and IR when compared to HC, indicating a Th2-mediated inflammatory process.

### Frequency of NKG2D+ cells in intestinal lamina propria NK cells in mice

**Figure 3:**
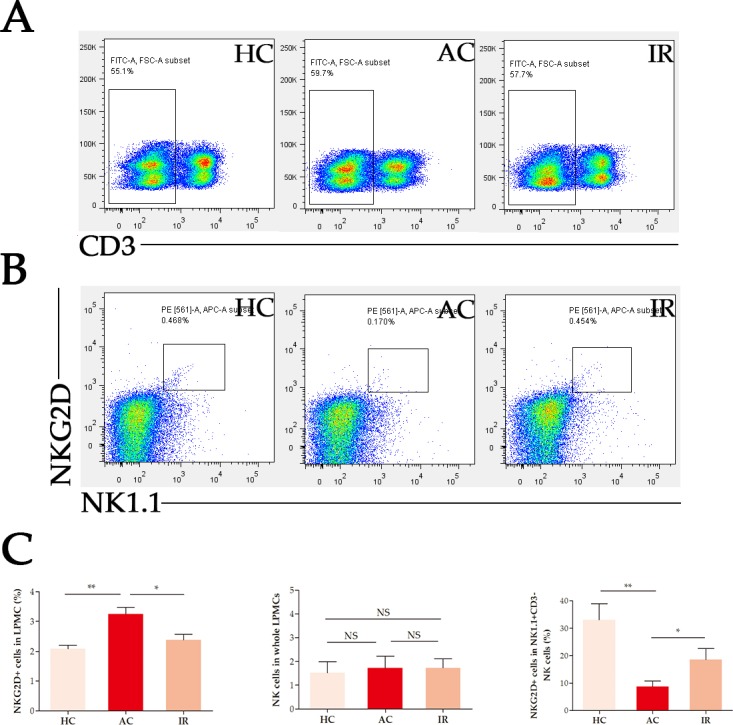
Frequency of NKG2D+ NK1.1+ CD3- cells in intestinal LPMCs **(A)** Frequency of CD3- cells in LPMCs. **(B)** Frequency of NKG2D+ NK1.1+ cells in CD3- LPMCs. **(C)** Statistics. HC: healthy controls, AC: acute colitis, IR: in remission, LPMCs: lamina propria mononuclear cells, ^*^: *P* < 0.05, ^**^: *P* < 0.01, ^***^: *P* < 0.001, NS: not significant.

In flow cytometric analysis, we found the frequency of NKG2D+ cells in LPMCs increased significant in AC compared to HC, and decreased significantly when the mice reached remission (Figure 3). The frequency of NK cells (NK1.1+ CD3+) made no significant change during the colitis. The frequency of NKG2D+ cells in lamina propria NK cells decreased significantly in AC, and increased significantly when mice reached remission, indicating a protective role of lamina propria NKG2D+ NK cells.

### Frequency of mucosal NKG2D+ NKp46+ NK cells in patients with UC

**Figure 4:**
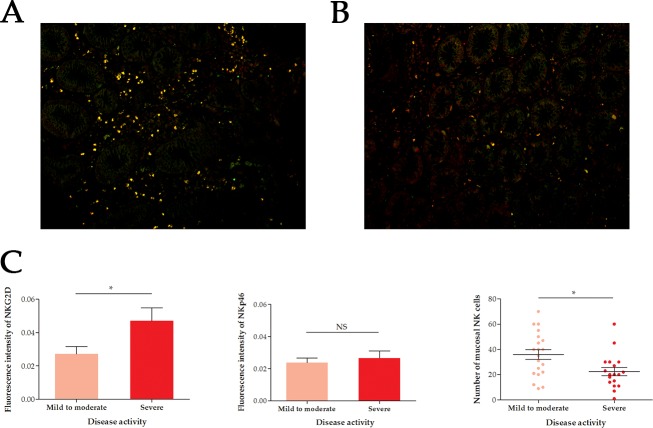
Immunofluorescence of mucosal NKG2D+ NKp46+ NK cells in UC patients of different disease activity **(A)** Mild to moderate ulcerative colitis (UC). **(B)** Severe UC. **(C)** Statistics. Image magnification was 400-fold. Red light: NKG2D, Green light: NKp46, Yellow light: NKG2D and NKp46, UC, ulcerative colitis, ^*^: *P* < 0.05, ^**^: *P* < 0.01, ^***^: *P* < 0.001, NS: not significant.

Immunofluorescence test was conducted to detect the frequency of mucosal NKG2D+ NKp46+ NK cells in UC patients. Compared with mild to moderate UC, the fluorescence intensity of NKG2D in intestinal mucosa was significantly higher in severe UC (*P* = 0.0389), while no significant difference was detected for NKp46 (*P* = 0.6004) (Figure 4). Furthermore, a higher frequency of NKG2D+ NKp46+ NK cells was found in the intestinal mucosa of severe UC than in mild to moderate UC (*P* = 0.0132).

### Cytokine secretion by human NK-92 cell line after MICA stimulation

NK-92 cell line was stimulated by soluble MICA at different concentrations (0, 2 and 10 pg/ml). The mRNA level of Th1 cytokines (TNF-α, IFN-γ) was remarkably higher than Th2 (IL-4, IL-6, IL-10) and Th17 (IL-17) cytokines when the cell line was stimulated by 10 pg/ml MICA (Figure 2B).

### Bioinformatics analysis of the role of TNF in UC and Crohn's colitis (CDc)

**Figure 5:**
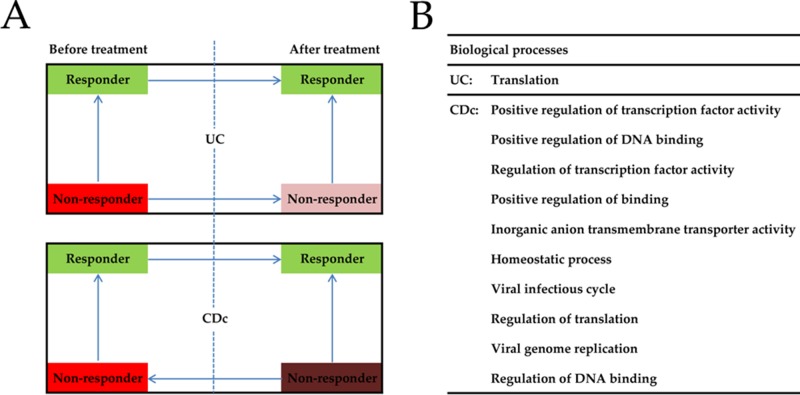
Bioinformatics analysis of the role of TNF in UC and CDc **(A)** The mRNA level of tumor necrosis factor in intestinal mucosa of UC and CDc before and after infliximab treatment. **(B)** Gene set enrichment analysis of biological processes related with TNF expression in UC and CDc (only list top 10). Responder: responder to infliximab treatment, Non-responder: non-responder to infliximab treatment; →: from the high mRNA level of TNF to the low level, TNF: tumor necrosis factor, UC: ulcerative colitis, CDc: Crohn's colitis.

Before anti-TNF treatment, TNF levels in UC non-responders were significantly higher than in responders (*P* = 0.006), and decreased significantly after treatment (*P* = 0.043) (Figure 5A; Supplementary Table 1). After anti-TNF treatment, TNF levels in CDc non-responders were significantly higher than in responders (*P* < 0.001) and before treatment (*P* = 0.001). The results indicated that TNF might play different roles in UC and CD.

1346 genes were differentially expressed between the groups divided by TNF expression levels in UC, while 266 DEGs were identified in CDc. UC and CDc were obviously different in DGEs distribution (*P* < 0.001 for χ^2^ test). In GSEA analysis, 194 gene sets were enriched in the CDc with TNF highly expressed, while only 1 in the UC (Figure 5B). This indicated that TNF in CD might be involved in more biological processes than in UC, and blocking TNF in CD might have a greater influence than in UC.

## DISCUSSION

In this study, we found that lamina propria NKG2D+ NK cells significantly decreased in active UC, and secreted Th1 cytokines (TNF and IFN-γ) after MICA stimulation. Considering that UC was characterized by a Th2 disorder, NKG2D+ NK cells might play a regulatory role by secreting Th1 cytokines in UC. However, it was controversial with the current opinions, and TNF had been regarded as a therapeutic target in both UC and CD. We guessed that the role of lamina propria NKG2D+ NK cells might be double-edged. Bioinformatics analysis also indicated different roles of TNF in UC and CD. TNF might participate in more biological processes in CD than UC, and blocking TNF probably had a greater influence in CD than UC, including efficacy and side effects. In previous meta-analyses, anti-TNF agents also showed much better efficacy in treating CD than UC [10, 11]. Furthermore, for side effects, anti-TNF therapy could significantly increase the incidence of infection in CD rather than UC [12, 13].

CD was a predominately Th1 and Th17 mediated process, and as an inflammatory and Th1 cytokine, there was no doubt that TNF played a pro-inflammatory role in CD. Previous studies also proved more effective of anti-TNF treatment in CD than UC. On the other hand, after anti-TNF discontinuation for 1 year, CD had a higher relapse rate than UC (40% vs. 28%) [14]. Furthermore, more UC patients could reach and maintain remission by non-specific anti-inflammatory drugs, like NSAIDs, corticosteroids and immunosuppressants, of which some showed similar efficacy with anti-TNF agents [15, 16]. However, for CD, anti-TNF agents were the most effective medications to induce and maintain remission, and prevent recurrence [17, 18]. All these indicated the double-edged role of TNF in UC, compared to CD.

The role of NK cells in the pathogenesis of IBD was not well illustrated, and different subtypes showed anti-inflammatory or pro-inflammatory effects [19]. In active UC, peripheral NK cells decreased significantly (*P* < 0.01) compared to inactive UC, while active CD had a higher proportion of NK cells than active UC (*P* < 0.01) [20]. After anti-TNF treatment, peripheral NK cells in responsive IBDs were significantly higher than non-responsive UC (*P* < 0.05) [21, 22]. These findings indicated that NK cells might played a regulatory role in the pathogenesis of UC. In CD, intestinal propria lamina NK cells were increased significantly compared with UC or HC (*P* < 0.05), but no significant difference was found between UC and HC, which was consistent with our results [6]. Nevertheless, as one source of Th1 cytokines, we thought some subsets of NK cells might be involved in UC.

NKG2D was one of main activated receptors on NK cells, which was also widely distributed on macrophages and T cells. The interaction of NKG2D and its ligand MICA was related with the pathogenesis of IBD [8, 9]. In CD, the percentage of mucosal and peripheral CD4+ T cells expressing NKG2D increased (*P* = 0.003; *P* = 0.01). It made no obvious change in UC (*P* > 0.05), but in peripheral blood, it was significantly lower in UC than CD (*P* = 0.003) [23]. In CD which was a predominately Th1 and Th17 mediated process, NKG2D was a functional marker of CD4+ T cells producing IL-17 and promoted intestinal inflammation, and this could be also used to illustrate the relatively low level of NKG2D+ CD4+ T cells in UC which was characterized by a Th2 process [24]. On the other hand, there was no difference in the percentage of mucosal and peripheral CD8+ T cells expressing NKG2D among UC, CD and HC. Moreover, in UC, peripheral γ/δ T cells expressing NKG2D was increased compared with CD (*P* < 0.05), while there was no difference in NKG2D+ CD56+ T cells [9]. These findings indicated that the role of NKG2D+ cells varied from immunocytes, tissues and diseases. Thus, some mucosal immunocyte subsets expressing NKG2D might play a regulatory role in UC. Furthermore, the decrease of LP NKG2D+ NK cells could also illustrate the high incidence of intestinal carcinogenesis in UC [25].

In conclusion, our study demonstrated the regulatory role of intestinal propria lamina NKG2D+ NK cells through secreting Th1 cytokines to regulate the Th1/Th2 imbalance in UC, despite of the concomitant pro-inflammatory effects of Th1 cytokines.

## MATERIALS AND METHODS

### Mice

Male BALB/c mice (6~8 weeks old) were purchased from the Experimental Animal Center of Wuhan University. To induce experimental colitis, mice were orally administered 4.5% DSS (dextran sulphate sodium, MP Biomedicals, USA) in distilled water for 7 days, while the controls were gave distilled water. Mice were humanely sacrificed if they reached a predetermined experimental end point (i.e. 7-day DSS administration for acute colitis and subsequent 7-day distilled water for remission).

### Evaluation of colitis severity

Colitis severity in mice was graded according to the body weight, stool consistency and hematochezia which were examined every other day. For those without macroscopic hematochezia, fecal occult blood test (Jiancheng Biotechnology Co., Ltd, China) was conducted. DAI (disease activity index) score was used to assess the disease activity in DSS-induce mice colitis (Table 1), and the colon length was measured after autopsy [26]. 5-μm sections of paraffin embedding were cut and stained with hematoxylin and eosin for histological assessment.

**Table 1:** Scoring system for disease activity index (DAI) in the study

Score	Weight loss (%)	Stool consistency	Hematochezia
0	0	Normal	Occult blood negative
1	1-5	Normal	Occult blood negative
2	6-10	Loose stool	Occult blood positive
3	11-15	Loose stool	Occult blood positive
4	>15	Diarrhea	Macroscopic blood

### ELISA assays

Tumor necrosis factor alpha (TNF-α), interferon gamma (IFN-γ), interleukin 4 (IL-4), IL-6, IL-10 and IL-17 were assayed by antibody sandwich ELISA in colonic lysates using a mouse ELISA immunoassay kit (Elabscience Biotechnology Co., Ltd, China) in accordance with the manufacturer's protocol.

### Isolation of intestinal lamina propria mononuclear cells (LPMCs)

LPMCs suspension was obtained as previously described [27]. The colons were flushed and opened longitudinally before cutting into 0.5~1.0 cm pieces. To remove epithelial cells and intraepithelial lymphocytes, colonic pieces were incubated in HBSS (Ca^2+^-/Mg^2+^-) containing 2 mmol/L EDTA, 10 mmol/L HEPES, 1 mmol/L DTT and 5% FCS twice for 15 min at 37°C under slow rotation. Then, the pieces were digested in HBSS (Ca^2+^+/Mg^2+^+) containing 5% FCS, 1.5 mg/ml Collagenase VIII (Sigma-Aldrich, USA) and 0.1 mg/ml DNase I (Thermo Scientific, USA) for 45 min at 37°C under slow rotation. The LPMCs were purified using 40% and 80% (Sigma-Aldrich, USA) Percoll gradients by centrifuging at 1000 g for 20 min.

### Flow cytometry

To detect NKG2D+ NK cells in LPMCs, cells were stained with APC-conjugated anti-mouse CD314 (NKG2D) antibody (eBioscience, USA), PE-conjugated anti-mouse NK1.1 antibody (eBioscience, USA), and FITC-conjugated anti-mouse CD3 antibody. For anti-NKG2D and anti-NK1.1 antibodies, related isotype controls were used as negative control. All flow cytometric analyses were performed on a FACS Calibur (BD Biosciences, USA), and the data were analyzed by FlowJo 7.6 (Treestar, Ashland, OR, USA).

### Patients' specimens and immunofluorescence

Intestinal mucosal specimens were endoscopically obtained from 20 patients with mild to moderate UC and 18 patients with severe UC, and only actively inflamed biopsies were obtained. The severity was defined by endoscopic and histological diagnoses from senior physicians in the departments of gastrointestinal endoscopy and pathology. Characteristics of the patients were shown in Table 2. For the detection of NKG2D+ NK cells in intestinal mucosa, samples were stained with anti-human NKG2D antibody (rabbit-derived, Bioss Biotechnology Co., Ltd, China), anti-human NKp46 antibody (mouse-derived, R&D Systems, USA), and subsequent secondary antibodies of Cy3-conjugated anti-rabbit IgG antibody (goat-derived, BOSTER Bioengineering Co., Ltd, China) and anti-mouse IgG antibody (goat-derived, BOSTER Bioengineering Co., Ltd, China). Immunofluorescent images were obtained under fluorescence microscopy (Olympus IX53, Japan), and fluorescence intensity was analyzed by Image-Pro Plus 5.0 (Media Cybernetics, Inc., USA). The number of mucosal NKG2D+ NK cells was measured by manual counting.

**Table 2:** Characteristics of included patients with ulcerative colitis

Variable	Disease activity		*P* value
	Mild to moderate	Severe	
Total cases	20	18	-
Age	39.7 ± 12.7	40.4 ± 11.4	0.8622
Sex (male)	14 (70%)	9 (50%)	0.2080
Disease location^#^			
Proctitis	8 (40%)	0 (0%)	
Left-sided colitis	10 (50%)	2 (11%)	< 0.0001
Extensive colitis	2 (10%)	16 (89%)	
ESR (mm/h)	11.1 ± 13.9	50.0 ± 28.1	< 0.0001
CRP (mg/L)	4.2 ± 5.0	48.7 ± 41.4	< 0.0001

UC: ulcerative colitis. ESR: erythrocyte sediment rate. CRP: C-reactive protein. ^#^: according to the Montreal classification (2012).

### Cytokine secretion by human NK-92 cell line after MICA stimulation

NK-92 cell line (granted by Professor Feili Gong, Tongji Medical College of Huazhong University of Science and Technology, Wuhan, China) was cultured in the α-MEM (Gibco, USA) supplemented with 12.5% FCS (Gibco, USA), 12.5% horse serum (HyClone, USA), and 100~200 U/ml recombinant human IL-2 (Shandong Quangang Pharmaceutical, Co., Ltd, China). Soluble MICA (Sino Biological Inc., China) was coated at different concentrations (0, 2 and 10 pg/ml) on 96-well plates, and NK-92 cell line was incubated for 24 hours. Total RNA was extracted, and synthesized into cDNA as previously described [10]. The cytokines were quantified by quantitative real-time polymerase chain reaction (qRT-PCR) using SYBR Green Master Mix (Toyobo, Japan). The human gene-specific primers were listed in Supplementary Table 2.

### Bioinformatics analysis of the role of TNF in UC and Crohn's colitis (CDc)

Gene expression raw data and related clinical data were downloaded from Gene Expression Omnibus (GEO) database (http://www.ncbi.nlm.nih.gov/geo/). Dataset GSE16879 studied the effect of infliximab treatment (anti-TNF monoclonal antibody) on mucosal gene expression profiles in UC and CDc patients (Supplementary Tables 3 and 4), and defined infliximab response according to the endoscopic and histologic findings. Raw expression data were calculated following the pre-processing procedures: (1) RMA background correction; (2) log_2_ transformation; (3) quantile normalization; (4) median-polish probeset summarization using the “affy” R package [28]. The expression value of TNF (probe: 207113_s_at) was extracted for differential analyses between groups.

Furthermore, in both datasets, samples were divided into two groups according to the expression level of TNF. To identify potential biological processes associated with CD or UC, gene set enrichment analysis (GSEA) was conducted to detect whether a series of priori defined biological processes were enriched in the gene rank derived from differentially expressed genes (DEGs) between the two groups. *P* value < 0.05 and false discovery rate (FDR) < 0.25 were chosen as the cut-off criteria.

### Statistical analysis

Statistical analysis was conducted using GraphPad Prism 5 (GraphPad Software, Inc, San Diego, CA). For dichotomous data, *χ^2^* test or Fisher's exact test was performed with SPSS Statistics V21.0 (IBM, USA). *P* value < 0.05 was considered statistically significant.

## SUPPLEMENTARY MATERIALS FIGURES AND TABLES


