# Supplementary material for: Regulatory role of NKG2D+ NK cells in intestinal lamina propria by secreting double-edged Th1 cytokines in ulcerative colitis
Source: Oncotarget. 2017 Oct 30;8(58):98945–52. doi: 10.18632/oncotarget.22132 (PMC5716779; doi:10.18632/oncotarget.22132)
Supplement: Supplementary file 1 [file oncotarget-08-98945-s001.pdf]

Regulatory role of NKG2D+ NK cells in intestinal lamina propria by secreting double-edged Th1 cytokines in ulcerative colitis

SUPPLEMENTARY MATERIALS

Supplementary Table 1: Statistics in bioinformatics analysis

Disease	Contrast	log_2(FC)	*P* value
UC	Respond before T *vs.* Respond after T	0.02293617	0.9448677
	Non-respond before T *vs.* Non-respond after T	0.485895	0.04303153
	Non-respond before T *vs.* Respond before T	0.8178617	0.006393387
	Non-respond after T *vs.* Respond after T	0.3549029	0.2205784
CDc	Respond before T *vs.* Respond after T	0.2357143	0.4305145
	Non-respond before T *vs.* Non-respond after T	-1.358477	0.001053406
	Non-respond before T *vs.* Respond before T	0.5214819	0.1307461
	Non-respond after T *vs.* Respond after T	2.115674	5.61E-07

Respond: respond to infliximab treatment. Non-respond: non-respond to infliximab treatment. T: infliximab treatment. UC: ulcerative colitis. CDc: Crohn's disease. log_2(FC): log_2-based change fold of gene expression value.

Supplementary Table 2: The human gene-specific primer list in the study

Gene	PCR primer sequence (5'→3')	Size (bp)
TNF-α	F: TCAGAGGGCCTGTACCTCAT<br>R: GGAAGACCCCTCCCAGATAG	220
IFN-γ	F: TGACCAGAGCATCCAAAAGA<br>R: CTCGAAACAGCATCTGACTC	227
IL-4	F: GGCAGTTCTACAGCCACCAT<br>R: GTTGGCTTCCTTCACAGGAC	158
IL-6	F: TTCGGTCCAGTTGCCTTCTC<br>R: GCCTCTTTGCTGCTTTCACA	227
IL-10	F: GACTTTAAGGGTTACCTGGGTTG<br>R: TCACATGCGCCTTGATGTCTG	112
IL-17A	F: CCACCTCACCTTGGAATCTC<br>R: CCCACGGACACCAGTATCTT	220
β-actin	F: AGCGAGCATCCCCCAAAGTT<br>R: GGGCACGAAGGCTCATCATT	285

F: forward. R: reverse. PCR: polymerase chain reaction. bp: base pair. TNF: tumor necrosis factor. IFN: interferon. IL: interleukin.

Supplementary Table 3: The sample information of ulcerative colitis in GSE16879

Before treatment			After treatment		
**Sample ID**	**Description**	**Group**	**Sample ID**	**Description**	**Group**
GSM364633	UCR1_beforeT	Respond	GSM422963	UCR1_afterT	Respond
GSM364634	UCR2_beforeT	Respond	GSM422965	UCR2_afterT	Respond
GSM364635	UCR3_beforeT	Respond	GSM422967	UCR3_afterT	Respond
GSM364636	UCR4_beforeT	Respond	GSM422969	UCR4_afterT	Respond
GSM364637	UCR5_beforeT	Respond	GSM422971	UCR5_afterT	Respond
GSM364638	UCR6_beforeT	Respond	GSM422973	UCR6_afterT	Respond
GSM364639	UCR7_beforeT	Respond	GSM422975	UCR7_afterT	Respond
GSM364640	UCR8_beforeT	Respond	GSM422977	UCR8_afterT	Respond
GSM364641	UCNR1_beforeT	Non-respond	GSM422979	UCNR1_afterT	Non-respond
GSM364642	UCNR2_beforeT	Non-respond	GSM422981	UCNR2_afterT	Non-respond
GSM364643	UCNR3_beforeT	Non-respond	GSM422983	UCNR3_afterT	Non-respond
GSM364644	UCNR4_beforeT	Non-respond	GSM422985	UCNR4_afterT	Non-respond
GSM364645	UCNR5_beforeT	Non-respond	GSM422987	UCNR5_afterT	Non-respond
GSM364646	UCNR6_beforeT	Non-respond	GSM422989	UCNR6_afterT	Non-respond
GSM364647	UCNR7_beforeT	Non-respond	GSM422991	UCNR7_afterT	Non-respond
GSM364648	UCNR8_beforeT	Non-respond	GSM422993	UCNR8_afterT	Non-respond
GSM364649	UCNR9_beforeT	Non-respond	GSM422995	UCNR9_afterT	Non-respond
GSM364650	UCNR10_beforeT	Non-respond	GSM422997	UCNR10_afterT	Non-respond
GSM364651	UCNR11_beforeT	Non-respond	GSM422999	UCNR11_afterT	Non-respond
GSM364652	UCNR12_beforeT	Non-respond	GSM423001	UCNR12_afterT	Non-respond
GSM364653	UCNR13_beforeT	Non-respond	GSM423003	UCNR13_afterT	Non-respond
GSM364654	UCNR14_beforeT	Non-respond	GSM423005	UCNR14_afterT	Non-respond
GSM364655	UCNR15_beforeT	Non-respond	GSM423007	UCNR15_afterT	Non-respond
GSM364656	UCNR16_beforeT	Non-respond	GSM423009	UCNR16_afterT	Non-respond

UCR: infliximab responders of ulcerative colitis. UCNR: infliximab non-responders of ulcerative colitis. T: infliximab treatment.

Supplementary Table 4: The sample information of Crohn's colitis in GSE16879

Before treatment			After treatment		
**Sample ID**	**Description**	**Group**	**Sample ID**	**Description**	**Group**
GSM423010	CDcR1_beforeT	Respond	GSM423011	CDcR1_afterT	Respond
GSM423012	CDcR2_beforeT	Respond	NA	NA	NA
GSM423013	CDcR3_beforeT	Respond	GSM423014	CDcR3_afterT	Respond
GSM423015	CDcR4_beforeT	Respond	GSM423016	CDcR4_afterT	Respond
GSM423017	CDcR5_beforeT	Respond	GSM423018	CDcR5_afterT	Respond
GSM423019	CDcR6_beforeT	Respond	GSM423020	CDcR6_afterT	Respond
GSM423021	CDcR7_beforeT	Respond	GSM423022	CDcR7_afterT	Respond
GSM423023	CDcR8_beforeT	Respond	GSM423024	CDcR8_afterT	Respond
GSM423025	CDcR9_beforeT	Respond	GSM423026	CDcR9_afterT	Respond
GSM423027	CDcR10_beforeT	Respond	GSM423028	CDcR10_afterT	Respond
GSM423029	CDcR11_beforeT	Respond	GSM423030	CDcR11_afterT	Respond
GSM423031	CDcR12_beforeT	Respond	GSM423032	CDcR12_afterT	Respond
GSM423033	CDcNR1_beforeT	Non-respond	GSM423034	CDcNR1_afterT	Non-respond
GSM423035	CDcNR2_beforeT	Non-respond	GSM423036	CDcNR2_afterT	Non-respond
GSM423037	CDcNR3_beforeT	Non-respond	GSM423038	CDcNR3_afterT	Non-respond
GSM423039	CDcNR4_beforeT	Non-respond	GSM423040	CDcNR4_afterT	Non-respond
GSM423041	CDcNR5_beforeT	Non-respond	GSM423042	CDcNR5_afterT	Non-respond
GSM423043	CDcNR6_beforeT	Non-respond	GSM423044	CDcNR6_afterT	Non-respond
GSM423045	CDcNR7_beforeT	Non-respond	GSM423046	CDcNR7_afterT	Non-respond

CDcR: infliximab responders of Crohn's colitis. CDcNR: infliximab non-responders of Crohn's colitis. T: infliximab treatment. NA: information not available.
